# Effects of Face-Mask Use on Dry Eye Disease Evaluated Using Self-Reported Ocular Surface Disease Index Scores: A Cross-Sectional Study on Nurses in Saudi Arabia

**DOI:** 10.7759/cureus.33071

**Published:** 2022-12-28

**Authors:** Rawan A Alsulami, Reema Alotaibi, Ghadeer Alsulami, Razan Alharbi, Raghad Alamoudi, Nooran O Badeeb, Hanan Al Kadi

**Affiliations:** 1 Medicine, King Abdulaziz University, Jeddah, SAU; 2 Medicine, King Abdulaziz University Hospital, Jeddah, SAU; 3 Ophthalmology, University of Jeddah, Jeddah, SAU; 4 Physiology, King Abdulaziz University, Jeddah, SAU

**Keywords:** ocular irritation, covid-19, ocular surface disease, dry eye disorder, sars-cov-2

## Abstract

Background: Wearing face masks has been an essential part of healthcare workers’ lives since the coronavirus disease 2019 (COVID-19) pandemic. This study aims to determine the association between prolonged face mask-wearing and dry eye disorder (DED) among female nurses.

Methods: An online questionnaire-based cross-sectional study was conducted at King Abdulaziz University Hospital, Jeddah, Saudi Arabia, between May 2021 and February 2022. It covered sociodemographic data, conditions associated with ocular irritation, and questions related to mask-wearing duration. The Ocular Surface Disease Index (OSDI) survey was used to measure DED severity. Binary logistic regression analysis was done and Odd’s ratios (OR) with 95% confidence intervals (CI) were reported.

Results: A total of 266 female nurses responded to this study. The majority of the sample (71.1%) fell in the normal-mild DED category (OSDI 0-22), while (28.9%) were categorized as the moderate-severe DED category (OSDI >22). We found a significant independent association of dry eye disorder with wearing a mask for >6 hours/day (OR 2.066, 95% CI: 1.083-3.944). Other significant predictors of DED in this study were wearing corrective eyeglasses (OR 2.382, 95% CI: 1.296-4.376) and having rheumatoid arthritis (OR 17.289, 95% CI: 1.794-166.7).

Conclusion: Wearing a face mask for > 6 hours/day was significantly associated with moderate to severe DED among female nursing staff. Ophthalmologists should be aware of this adverse effect in order to promote ways to relieve this condition.

## Introduction

As the first line of defense during health crises, such as the coronavirus disease 2019 (COVID-19) pandemic, healthcare workers are often required to wear facemasks for prolonged periods [1]. Many of these individuals report symptoms of ocular irritation, dryness, and redness while wearing facemasks [2-5]. Yet, few studies have examined the association between prolonged facemask wearing and dry eye disease (DED) among healthcare professionals [5]. This study, therefore, aimed to assess the association between DED and prolonged facemask wearing among nurses working in a tertiary healthcare hospital.

## Materials and methods

We conducted this descriptive cross-sectional study in Jeddah, Saudi Arabia, between May 2021 and February 2022. Using Stata software (StataCorp., College Station, TX), we determined the required sample size at alpha 0.05 and power of 0.90 to be 184 subjects. Eligible participants included female nurses working at King Abdulaziz University Hospital. Participants with previous diagnoses of DED were excluded. Prior to starting the study, the questionnaire was piloted with a group of 30 physicians who did not participate as study subjects. As the online questionnaire was anonymous, consent was assumed through the completion of the questionnaire. We obtained approval from the research ethics committee with reference number 187-21.

With assistance from head nurses in each department, the online questionnaire was distributed randomly to 1,227 nurses. The questionnaire comprised 29 questions in three main sections covering personal and sociodemographic data, conditions associated with ocular irritation, and working hours and ocular irritation symptoms. The Ocular Surface Disease Index (OSDI) questionnaire, a validated tool for assessing symptoms of ocular irritation and DED, was included in the third section of the questionnaire. We used the OSDI total score to classify the respondent’s dry eye symptoms as normal-mild (0-22 points) and moderate-severe (23-100 points) [6].

Data were analyzed using the Statistical Package for Social Sciences version 20.0 (IBM Corp, Armonk, NY). Descriptive data are expressed as frequencies and proportions. We analyzed differences between categorical variables using the Chi-square test. Logistical regression was used to analyze factors independently associated with ocular irritation and dryness. We considered a P-value of <0.05 to be statistically significant.

## Results

Of the 266 female nurses who completed the online survey, half (50.8%) were younger than 40 years old, and most worked in the pediatric (34.2%) and emergency (19.2%) departments. Only eight participants used contact lenses (3.0%), compared to 34.2% who reported wearing corrective eyeglasses. Regarding chronic conditions affecting the eyes, 5.6% reported having allergic conjunctivitis, 2.6% had rheumatoid arthritis, 0.4% had Sjogren’s syndrome, and none had glaucoma. Table 1 summarizes the results.

**Table 1:** Characteristics of study participants

Variable	Categories	Number	%
Age	<40 years	135	50.8%
	>=40 years	131	49.2%
Marital status	Married	213	80.1%
	Single	53	19.9%
Corrective eyeglasses	Yes	91	34.2%
	No	175	65.8%
Contact lenses	Yes	8	3%
	No	258	97%
Allergic conjunctivitis	Yes	15	5.6%
	No	251	94.4%
Sjogren’s syndrome	Yes	1	0.4%
	No	265	99.6%
Rheumatoid arthritis	Yes	7	2.6%
	No	259	97.4%
Department	Internal medicine	4	1.5%
	Obstetrics and gynecology	15	5.6%
	Emergency	51	19.2%
	Pediatrics	91	34.2%
	Others	105	39.5%

Only 38 (14.3%) nurses reported taking regular medications. None used eye drops regularly. More than half (58.6%) worked at least 40 hours per week. Regarding face mask-wearing, 161 nurses (60.5%) reported wearing a face mask for at least six hours per day. Of those, more than a third (35.4%) were considered to have a moderate-severe DED (OSDI >22). Most (71.1%) were classified as normal-mild DED category (OSDI 0-22), and 28.9% as moderate-severe (OSDI >22). Table 2 summarizes the univariate analysis of different risk factors and their associations with DED.

**Table 2:** Univariate analysis of risk factors and their associations with dry eye disease (DED) Abbreviations: *DED: Dry Eye Disease

Risk factor	Category	Total	Normal		DED*		OR	95% CI		P-value
			N	%	N	%				
Duration of wearing a mask	6 hr/day	105	85	81.0%	20	19.0%	2.329	1.298	4.179	0.004
	≥6 hr/day	161	104	64.4%	57	35.4%				
Age	<40 years	135	91	67.4%	44	32.6%	0.696	0.408	1.188	0.183
	≥40 years	131	98	74.8%	33	25.2%				
Duration of using mobile	<14 hr/week	79	53	67.1%	26	32.9%	0.764	0.433	1.350	0.354
	≥14 hr/week	187	136	72.7%	51	27.3%				
Duration of watching TV	<14 hr/week	237	169	71.3%	68	28.7%	1.118	0.485	2.579	0.793
	≥14 hr/week	29	20	69%	9	31%				
Duration of using computer	<14 hr/week	163	121	74.2%	42	25.8%	1.483	0.866	2.540	0.150
	≥14 hr/week	103	68	66%	35	34%				
Wearing corrective eyeglasses	No	175	136	77.7%	39	22.3%	2.500	1.445	4.325	0.001
	Yes	91	53	58.2%	38	41.8%				
Wearing contact lenses	No	258	184	71.3%	74	28.7%	1.492	0.348	6.402	0.694
	Yes	8	5	62.5%	3	37.5%				
Allergic conjunctivitis	No	251	181	72.1%	70	27.9%	2.263	0.791	6.473	0.144
	Yes	15	8	53.3%	7	46.7%				
Rheumatoid arthritis	No	259	188	72.6%	71	27.4%	15.887	1.879	134.297	0.003
	Yes	7	1	14.3%	6	85.7%				
Taking medications	No	228	165	72.4%	63	27.6%	1.528	0.743	3.139	0.246
	Yes	38	24	63.2%	14	36.8%				

The presence of moderate to severe DED based on OSDI score was significantly associated with wearing a mask for >6 hours/day (P=0.004, Odd’s ratio {OR} 2.066, CI 1.083-3.944), wearing corrective eyeglasses (P=0.001, OR 2.382, CI 1.296-4.376), and having rheumatoid arthritis (P=0.003, OR 17.289, CI 1.794-166.7). Age, number of hours using a mobile device or computer, number of hours watching television, wearing contact lenses, using medications, or having allergic conjunctivitis were not significantly associated with DED (P value <0.05 for all associations). Table 3 summarizes the results.

**Table 3:** Multivariate logistic regression of risk factors for dry eye disease

Risk factors	Categories	OR	95% CI		P- value
Duration of wearing a mask	6 hr/day	Reference	1.083	3.944	.028
	≥ 6 hr/day	2.066			
Wearing corrective eyeglasses	No	Reference	1.296	4.376	.005
	Yes	2.382			
Rheumatoid arthritis	No	Reference	1.794	166.661	.014
	Yes	17.289			

## Discussion

Prolonged use of face masks among health care practitioners is associated with conditions such as DED, which can degrade the quality of life and productivity [7]. Using the OSDI scoring system in this study, we found that wearing face masks for more than six hours per day was significantly and independently associated with moderate to severe DED. This result aligns with that of Aksoy and Simsek, who found significantly increased OSDI scores among those wearing face masks for more than eight hours per day [8]. Another study by Krolo et al. found that wearing a face mask for three-six hours per day was significantly associated with increased OSDI scores among study participants [9].

In contrast to our findings, a study by Boccardo L reported no significant association between mask-associated dry eye and self-reported dry eye symptoms [3], including foreign body sensation, dryness, irritation, and itching or burning. However, the study did not include a validated dry eye questionnaire, nor did it assess dry eye symptoms with defined durations of mask usage [3]. Another study by Al‐Dolat et al. in Jordan did not find a significant independent association between DED and wearing a face mask [10]. However, their study population included medical students in years 1-6 during the peak of the pandemic, when most of their academic activities were virtual, and only 6.9% of participants reported wearing a face mask for more than six hours per day [10], compared to 60.5% in our study, which focused on nursing staff who served as front-line health care workers during the pandemic.

The pathophysiological factors that may aggravate DED symptoms during prolonged face mask-wearing remain unclear. Many studies have suggested that the ocular surface is affected by the convection of air around the eyes caused by ill-fitting masks. A study by Arriola-Villalobos et al. objectively showed that face masks could cause tear film instability via an upward air-blowing mechanism, which accelerates tear evaporation [11]. Another study by Aksoy and Simsek found that wearing face masks causes a reduction in tear break-up time and Schirmer-1 measurements, which improves after securely taping the open upper portion of the mask to the face [8]. Results in a sample of patients with obstructive sleep apnea syndrome who use continuous positive airway pressure (CPAP) devices similarly indicate that the wearing of a CPAP mask causes eye irritation and tear evaporation due to air escaping through the mask [12]. Appliances that mechanically draft air over the face, such as certain air-purifying respirators, also are linked to an increase in dry eye symptoms [13]. In the current study, the effect of face masks on DED was noticeable after long-term, consistent, daily use rather than sporadic use.

Face masks may limit movement of the lower eyelid, leading to increased tear evaporation and aggravation of ocular surface disease symptoms [14]. Wearing masks also may increase carbon dioxide in the respiratory outflow, which in turn may decrease tear film pH and impair the ocular surface [12].

Climate and air temperature also affect the ocular surface, with warmer temperatures more likely to cause tear film evaporation. If the average air temperature inside the mask is 36-37°C and that warm air travels in an upward direction, it can accelerate the evaporation of tears [2,15]. This effect can be worsened in warmer climates, such as Jeddah in the Kingdom of Saudi Arabia, particularly if mask-wearing is mandatory even outdoors [16].

In our study, wearing corrective spectacles was significantly and independently associated with moderate to severe DED. Boccardo similarly reported that participants wearing glasses had a higher risk of mask-associated dry eye [3]. Entrapment of exhaled air between the posterior part of the eyeglasses and the anterior surface of the eye might lead to ocular surface irritation, DED symptoms, and ocular inflammation [2,17].

Our present study had several limitations. The subjective character of the OSDI score could have introduced bias into the results. The lack of objective testing to correlate the OSDI score with dry eye signs on clinical examination means that our results must be interpreted with caution. Including only female participants and focusing on one healthcare profession (i.e., nursing) limits the generalizability of our findings. Finally, there was no direct observation or questioning about mask fitting and the history of refractive surgery.

## Conclusions

Using the ODSI scoring system, this study identified a significant relationship between wearing face masks and moderate to severe DED among female nursing staff. This ocular surface disorder can have troubling symptoms that interfere with one’s work and everyday life. Ophthalmologists, thus, should be aware of the adverse effects of face mask wearing so that they can help promote ways to alleviate symptoms. Further studies on different populations and objective studies that relate DED symptoms to clinical signs also are necessary to improve patient outcomes.
